# Immunophenotypic profiles and prognosis for colorectal mucinous adenocarcinomas are dependent on anatomic location

**DOI:** 10.1002/cam4.5803

**Published:** 2023-03-14

**Authors:** Chirag Patel, Michael Behring, Sameer Al Diffalha, Deepti Dhall, Goo Lee, Chandrakumar Shanmugam, William E. Grizzle, Upender Manne

**Affiliations:** ^1^ Department of Pathology University of Alabama at Birmingham Birmingham Alabama USA; ^2^ O'Neal Comprehensive Cancer Center University of Alabama at Birmingham Birmingham Alabama USA; ^3^ Department of Pathology RVM Institute of Medical Sciences & Research Center, KNR University of Health Sciences Siddipet India

**Keywords:** colorectal cancer, mucinous adenocarcinomas, non‐mucinous adenocarcinomas, prognostic biomarker, rectal cancers, tumor location

## Abstract

**Background:**

The prognostic value of mucinous adenocarcinomas (MCAs, exhibiting >50% extracellular mucin) of the colorectum, in relation to their anatomic location is not well studied.

**Materials and Methods:**

We compared MCAs (*n* = 175) with non‐MCAs (NMCAs, *n* = 1015) and the cancer‐specific survival rates were evaluated, based on their anatomic site, by univariate Kaplan–Meier and multivariate Cox methods. Subsets of these tumors were immunostained for MUC1, MUC2, Bcl‐2, and p53.

**Results:**

MCAs were more commonly found in the right colon, were of high‐grade, and were more prevalent in younger patients (<40 years). They exhibited strong expression of MUC2 and Bcl‐2 and showed less p53 nuclear staining. In contrast, most NMCAs were low‐grade with high expression of MUC1. MCAs of the rectum were associated with poorer outcomes relative to NMCAs (HR 1.85, CI 95% 1.15–2.97), even though the distributions of advanced‐stage tumors were similar.

**Conclusion:**

Late‐stage disease and age were poor independent prognostic indicators of cancer‐specific deaths across all tumor locations. In summary, rectal MCAs have a poor prognosis.

## Graphical abstract

Overall, our study found that mucinous adenocarcinomas (MCAs) were more commonly located in the right colon, high grade, and prevalent in younger patients (<40 years). They exhibited strong expression of MUC2 and Bcl‐2 and showed less aberrant p53 nuclear staining. In adjusted multivariate survival models split by tumor location, MCAs of the rectum were associated with poorer outcomes relative to NMCAs (HR 1.85, CI 95% 1.15‐2.97). Our results support the concept that patients with MCAs located in the rectum should be considered for rigorous treatment regimens.

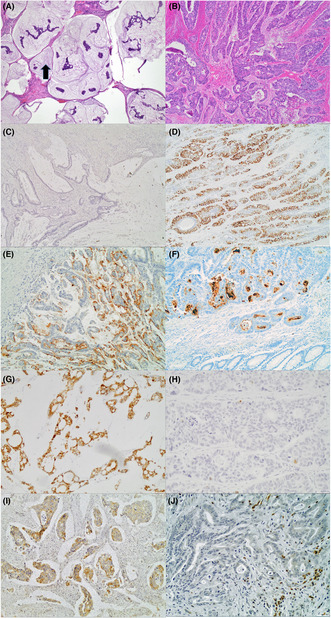

## 1 INTRODUCTION

Colorectal cancers (CRCs) were responsible for 1.9 million new cases in 2020, accounting for ~900,000 yearly deaths worldwide.^1^

Mucinous colorectal adenocarcinomas (MCAs) are defined by the extracellular mucin that accounts for ≥50% of the tumor volume.^2^ Of all CRCs, 10%–15% are MCAs.^2^ Currently, there is a debate regarding the prognosis for MCAs in comparison to non‐mucinous colorectal adenocarcinomas (NMCAs). Patients with MCAs exhibit poorer overall survival relative to those with NMCAs (48.4 months verss. 60.2 months).^3^ However, the overall survival rates for patients with MCAs are not significantly different from those with NMCAs when controlled for tumor stage (5‐ and 8‐year survival rates are 78.6% and 68.8% for MCAs and 72.3% and 63.8% for NMCAs).^4^ Findings on the prognostic importance of tumor location in the GI tract are inconsistent,^5^ and there is a scarcity of large studies examining site‐specific differences in the survival of these patients. In the present analysis, 175 MCAs were compared to 1015 NMCAs for demographic and clinicopathologic features and for the phenotypic expression of MUC1, MUC2, p53, and Bcl‐2. Cancer‐specific survival was evaluated to assess the effect of mucin status based on the tumor anatomic site after adjusting for confounding factors.

## 2 MATERIALS AND METHODS

### 2.1 Histopathological analysis

Formalin‐fixed, paraffin‐embedded (FFPE) blocks and hematoxylin and eosin (H&E)‐stained slides of 175 MCAs and 1015 NMCAs were collected from the Pathology Department of the University of Alabama at Birmingham and evaluated for mucin content. Slides and pathology reports from 1985 to 2005 were reviewed by three GI pathologists. This lengthy study period was selected because patients with early‐stage CRCs have longer survival. Tumors with >50% of extracellular mucinous components were classified as MCAs and the rest as NMCAs. Consecutive tissue sections, 5‐μM thick, were immunostained for MUC1, MUC2, Bcl‐2, and p53, for subsets of cases/available tissues, to demonstrate phenotypic expression patterns and status for descriptive/correlative purposes. Staining evaluations were performed as described in our prior studies.^6^, ^7^ In brief, the intensity of immunostaining of individual cells was scored on a scale from 0 (no staining) to 4 (strongest intensity). In addition, each pathologist estimated the proportion of cells stained at each intensity. The percent of cells at each intensity was multiplied by the corresponding intensity value to obtain an immunostaining score that ranged from 0 to 4. The scores of all pathologists were combined to obtain an overall average immunostaining score. For cytoplasmic staining, based on the staining intensity of a marker in normal/benign tissue, immunostaining scores were chosen to be positive for these markers. The Score of ≥0.5 for Bcl‐2 and ≥10% nuclear positivity for p53 nuclear accumulation, without antigen retrieval, ≥50% nuclear positivity with antigen retrieval, was chosen. However, a cutoff value of the immunostaining score of ≥0.5 plus at least 25% of tumor cells immunostaining, after antigen retrieval, was required to classify a tumor as positive for expression of either the MUC1 or MUC2 antigens. Immunohistochemical markers were dichotomized into positive (overexpressed/high) and negative (downregulated/low) categories using the median expression in normal tissue as the cut‐point.^6^, ^7^

### 2.2 Design and patient inclusion criteria

We employed a retrospective cohort design with death from cancer as the primary outcome. Patients who lacked vital status at follow‐up, died within 1 month of follow‐up, or had previous or concurrent cancers, were excluded. Demographic variables were sex, age at the time of diagnosis, and self‐reported race. Pathologic variables including AJCC‐TNM stage, tumor location, differentiation/grade (per current WHO guidelines), and death due to cancer, were recorded. Follow‐up data for patients were collected from 1985 through 2014. Time to CRC‐related death was calculated from the date of surgery.

### 2.3 Statistical analyses

The univariate analysis for MCA/NMCA groups was performed with chi‐square testing for categorical variables and *t*‐tests were performed for continuous variables. The statistical significance of *p*‐values was 0.05. Additional univariate analyses of tumor location‐stratified subsets (proximal colon, distal colon, and rectum) were performed. Kaplan–Meier plots and log‐rank tests were used for the determination of univariate survival. Analyses of adjusted survival in stratified MCA‐only and NMCA‐only groups were performed by Cox proportional hazard models.

## 3 RESULTS

### 3.1 Overall differences by mucin status

MCAs were more commonly found in the right colon (86 of 173, 50%); were high‐grade (26% MCAs vs. 17% NMCAs); were more prevalent in younger patients (<40 years, MCAs 8% vs. NMCAs 4%); exhibited stronger expression of MUC2 (76 of 77, 99%) and Bcl‐2 (49 of 165, 30% vs. NMCAs 220 of 962, 23%); and had a lower number of nuclear p53 positive malignant cells (MCAs 33 of 90, 37% vs. NMCAs 266 of 496, 54%). In contrast, most NMCAs were low‐grade tumors and positive for MUC1 (NMCAs 148 of 290, 51%, vs. MCAs 29 of 80, 36%) (Table 1). There were no differences in relation to gender, race, or older age (not shown). In tumor location‐based analyses, MUC2 expression was higher in MCAs across all sites and p53 was lower only in distal MCAs (not shown).

**TABLE 1:** Comparison of pathological and demographic features of mucinous/non‐mucinous carcinomas.

	Mucinous (*N* = 175)	Non‐mucinous (*N* = 1015)	*p*‐value
Demographic			
Age (years)	64.1 ± 14.4	64.2 ± 12.5	0.919
Age (0–40)	14 (8.1%)	39 (3.9%)	0.027
*Race*			0.862
Caucasian (CA)	107 (61%)	631 (62.2%)	
African American (AA)	68 (38.9%)	384 (37.8%)	
*Gender*			0.549
Female	84 (48.0%)	459 (45.2%)	
Male	91 (52.0%)	556 (54.8%)	
Pathology^a^			
*Tumor location*			0.151
Proximal	86 (49.7%)	424 (41.8%)	
Distal	50 (28.9%)	344 (33.9%)	
Rectum	37 (21.4%)	246 (24.3%)	
*AJCC stage* ^a^			0.428
I	27 (15.5%)	210 (20.9%)	
II	66 (37.9%)	252 (35.0%)	
III	57 (32.8%)	305 (30.3%)	
IV	24 (13.8%)	140 (13.9)	
*Grade* ^a^			0.008
Low/Moderate	126 (73.6%)	836 (83.1%)	
High	45 (26.3%)	170 (16.9%)	
*MUC1* ^a^			0.027
Negative/low	51 (63.8%)	142 (49.0%)	
Positive/high	29 (36.2%)	148 (51.0%)	
*MUC2* ^a^			<0.0001
Negative/low	1 (1.3%)	105 (42.5%)	
Positive/high	76 (98.7%)	142 (57.5%)	
*p53* ^a^			0.004
Negative/low	57 (63.3%)	230 (46.4%)	
Positive/high	33 (36.7%)	266 (53.6%)	
*Bcl‐2* ^a^			0.072
Negative/low	116 (70.3%)	742 (77.1%)	
Positive/high	49 (29.7%)	220 (22.9%)	
*Adjuvant therapy*			0.748
Missing^b^	40 (22.9%)	258 (25.4%)	
None	130 (74.3%)	732 (72.1%)	
Yes	5 (2.9%)	25 (2.5%)	

^a^ Data are not available for all cases.

^b^ Before 2000, adjuvant therapy was not common practice at UAB.

**FIGURE 1:**
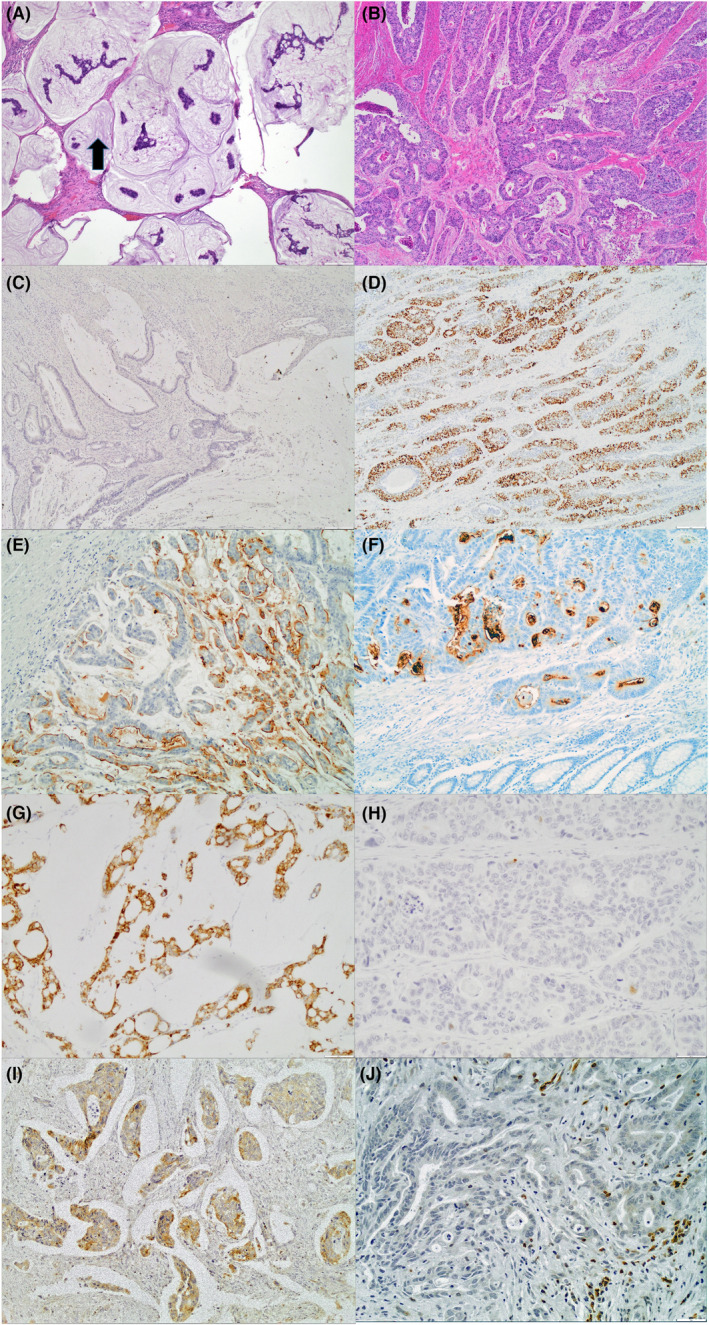
MCA with abundant extracellular mucin as shown by arrow (A, H&E, 4×); NMCA, which shows conventional adenocarcinoma without prominent mucin production (B, H&E, 4×); MCAs overall showed lower expression of nuclear p53 staining (C, p53, 4×) in contrast to the NMCAs, which showed higher expression of p53 (D, p53, 4×). MCAs showed slightly lower positive expression for MUC1 (E, MUC1, 4×) in contrast to NMCAs (F, MUC1, 4×). MCAs showed consistently higher expression for MUC2 (G. MUC2, 10×) in comparison to NMCAs, which showed lower expression (H, MU2, 20×); MCAs expressed slightly higher positive expression for BCL2 (I, BCL2, 4×) as compared to NMCAs (J, BCL2, 20×).

Histologic types and immunostaining patterns are shown in Figure 1. Intratumoral mucin patterns are shown in Figure 1A, B. NMCAs exhibited more nuclear p53 staining (Figure 1D), relative to MCAs (Figure 1C), and were more often positive for MUC1 (Figure 1F) than MCAs (Figure 1E). In contrast, MCAs had higher positive expression for MUC2 (Figure 1G).

### 3.2 Univariate survival by mucin status

**FIGURE 2:**
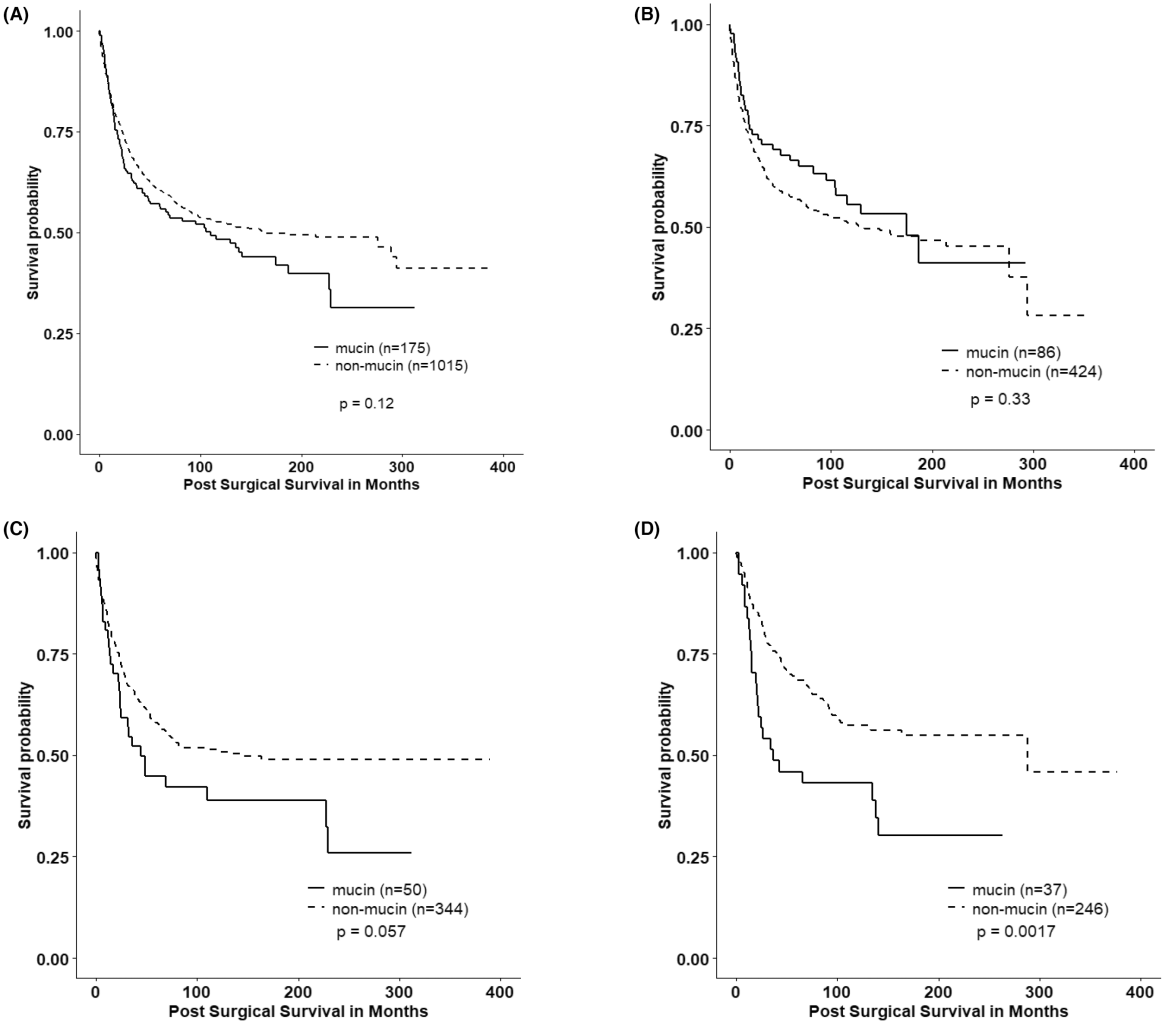
Univariate survival analyses by mucinous/non‐mucinous histological status in (A) the complete cohort (all locations); (B) proximal tumors (14% MCAs); (C) distal tumors (11% MCAs); and (D) rectal tumors (12% MCAs).

In univariate analyses, there was no difference in cancer‐specific survival between those with MCAs and those with NMCAs (*p* = 0.12) when all anatomic locations were grouped together (Figure 2A). For proximal colon tumors, mucinous histology had no appreciable effect on cancer‐specific survival (Figure 2B); the distributions of advanced staged tumors were similar (χ^2^, *p* = 0.787). Furthermore, a similar, although not statistically significant, finding of poor survival was evident for distal tumors (*p* = 0.057) (Figure 2C), and MCAs in the rectum showed poor outcomes in comparison to NMCAs (log rank, *p* = 0.0017) (Figure 2D).

### 3.3 Adjusted survival models based on tumor location

An overall model of cancer‐specific survival adjusted for age and stage showed an interaction between mucin status and tumor anatomic site; patients with MCAs and rectal tumors had a 2.1 times greater hazard of death from CRC (CI = 1.2–3.8), independent of age, stage, gender, and race (data not shown). Since the interaction between tumor location and mucin status was significant, we performed additional analyses in subsets of data for proximal colon, distal colon, and rectal tumors according to the mucin status. For rectal tumors, the adjusted hazard of death was 1.85 times greater for MCA tumors than for NMCAs (CI = 1.15–2.97). The mucin status of tumors of the proximal and distal colon was not a predictor of cancer‐specific survival as determined in age/race/gender/stage‐adjusted models (Table 2).

**TABLE 2:** Adjusted cancer‐specific survival models stratified by tumor location.

		Proximal tumors (*n* = 614)			Distal tumors (*n* = 454)			Rectal tumors (*n* = 316)		
Variable	Comparison	HR	95% CI	*p*‐value	HR	95% CI	*p*‐value	HR	95% CI	*p*‐value
Age	*Continuous by year*	1.01	1.00–1.02	0.009	1.01	1.00–1.02	0.063	1.02	1.00–1.03	0.03
Race	*Caucasian* vs. *African American*	0.86	0.66–1.12	0.26	0.98	0.73–1.33	0.9	1.11	0.71–1.73	0.64
Gender	*Male* vs. *female*	0.93	0.72–1.21	0.6	0.88	0.66–1.19	0.41	1.28	0.88–1.87	0.2
Stage	*Late* (*III&IV*) vs. *early* (*I&II*)	4.07	3.08–5.4	<0.001	3.12	2.29–4.26	<0.001	3.09	2.12–4.5	<0.001
Grade	*High* vs. *low/moderate*	2.03	1.52–2.71	<0.001	1.48	0.98–2.23	0.06	1.35	0.88–2.08	0.17
Mucin	*Mucinous* vs. *non‐mucinous*	0.89	0.63–1.27	0.46	1.12	0.79–1.82	0.39	1.85	1.15–2.97	0.01

## 4 DISCUSSION

The colorectum is frequently considered a single organ in that the epithelium of the proximal and distal colon and the rectum appears similar. However, there are various biological and genetic differences.^8^ Cancers in the colon and rectum differ clinically, as rectal cancers have a greater rate of local recurrence and are treated differently.^9^ The present study demonstrated that MCAs located in the rectum exhibit a worse prognosis relative to NMACs.

Our finding of the importance of rectal location in relation to the survival of patients with MCAs agrees with the results of an epidemiologic study of CRCs.^10^ Additionally, a meta‐analysis of 8 studies of rectal cancer found worse overall survival, a reduced rate of pathological complete response, less tumor down‐staging after therapy, and higher rates of positive margins in MCA rectal tumors compared to NMCAs.^11^ A Korean study found site‐specific survival of patients with MCA as an independent prognostic factor only for colon cancers.^12^ However, for their cohort of 6475 patients, this might be due to the higher frequency of MCAs in proximal (9%) versus rectal (2%) locations. Our observations of MCA frequency are consistent with prior observations of 10%–15% overall,^2^ and 8%–12% or 3%–7% in the colon and rectum, respectively.^5^ In contrast, a population‐based European study of only colon cancer patients (>120,000) found that MCAs do not affect survival.^13^

For CRCs, tumor anatomic site, stage, and molecular profiles, specifically microsatellite instability (MSI), *KRAS, BRAF,* and *PI3K*, aid in designing treatments. However, histologic types (MCAs vs. NMACs) are relevant entities because they exhibit distinct molecular profiles. Patients with stage III or IV MCAs, compared with NMACs, exhibit higher expression of MUC2 and Muc5AC and mutations in genes of the Ras/Raf/MAPK and PI3K/Akt/mTOR pathways; those with high MSI are poor responders to chemotherapy.^14^ However, the molecular determinants that contribute to the MCA phenotype and their prognostic implications are not understood.

In the present study, we found that MUC2 was overexpressed in almost all MCAs; its expression was seen in the epithelial cells that comprise the MCAs as compared to the normal colon (Figure 1F) regardless of tumor location. Our prior study of MUC1 and MUC2 in CRCs showed that MUC1 is an indicator of poor prognosis for Caucasian patients, but there is, for African American or Caucasian patients, no prognostic value for MUC2 when colon and rectal cancers along with MCAs and NMCAs are combined.^7^ Other studies show that high expression of MUC2 in CRCs is associated with a favorable prognosis.^15^

Although our findings were adequately powered, they have a limitation. Since data for immunohistochemical markers were incomplete, we did not adjust the expression status of the markers in the survival models. Missing data also precluded treatment as a variable for analysis. Additionally, MSI status, an important prognostic marker, was not available for our patients. So, we could not study the effect of MSI status on the poor survival of rectal MCAs. Previous studies reported that MCAs in the proximal colon have a higher rate of MSI than in the distal colon and rectum. However, our findings that, in the rectum, the survival of MCAs was poorer than that of NMCAs need to be evaluated in larger studies in the context of MSI.

In sum, the current finding that MCAs located in the rectum is associated with a poor prognosis is clinically relevant given their distinct genetic/molecular differences. Since the therapeutic efficacy and overall survival for late‐stage patients with MCAs versus NMCAs are controversial, the current results support the concept that patients with MCAs located in the rectum should be considered for rigorous treatment regimens. Additionally, as almost all MCAs exhibit strong MUC2 expression regardless of tumor location, studies are needed to determine the link between MCAs and the poor prognosis for patients with rectal cancers and the association between MCAs and elevated levels of MUC2.

## AUTHOR CONTRIBUTIONS

**Chirag Patel:** Conceptualization (equal); data curation (lead); formal analysis (supporting); investigation (lead); project administration (equal); writing – original draft (lead); writing – review and editing (lead). **Michael Behring:** Conceptualization (equal); data curation (equal); formal analysis (lead); methodology (equal); visualization (equal); writing – original draft (lead); writing – review and editing (lead). **Sameer Al Diffalha:** Data curation (supporting); investigation (supporting); resources (equal); writing – review and editing (supporting). **Deepti Dhall:** Data curation (supporting); investigation (supporting); project administration (supporting); writing – review and editing (supporting). **Goo Lee:** Methodology (supporting); resources (supporting); writing – review and editing (supporting). **Chandrakumar Shanmugam:** Conceptualization (supporting); formal analysis (supporting); methodology (supporting); writing – original draft (supporting). **William E. Grizzle:** Data curation (supporting); resources (supporting); writing – review and editing (supporting). **Upender Manne:** Conceptualization (equal); funding acquisition (lead); supervision (lead); visualization (lead); writing – review and editing (equal).

## FUNDING INFORMATION

These studies were supported in part by institutional impact funds (Department of Pathology and School of Medicine of the University of Alabama at Birmingham, UAB) and from the National Institutes of Health (NIH)‐National Cancer Institute (NCI) (U.S.) award, 5U54CA118948 to UM.

## CONFLICT OF INTEREST STATEMENT

The authors declare no competing financial interests.

## ETHICS STATEMENT

The study was conducted in accordance with the Declaration of Helsinki, and approved by the Institutional Review Board of University of Alabama at Birmingham (UAB), Birmingham, AL. The title of the approval is, “*Prognostic Molecular Markers of Colorectal Cancer*” (approval # IRB‐020830005), for utilizing remnants of diagnostic CRC tissues. An informed consent waiver was granted by the IRB for this purpose.

## CONSENT FOR PUBLICATION

All authors have read and agreed to the final version of the manuscript.

## Data Availability

Original data are presented in this article and any questions can be directed to the corresponding author.
